# Knowledge, Attitudes, and Practices Regarding Infection Prevention and Control Among Australian Sonographers: A Cross-Sectional Survey

**DOI:** 10.3390/healthcare14213593

**Published:** 2026-10-23

**Authors:** Dania Abu Awwad, Katya Amadita, Sheri Anne Rae, Sarah Lewis, Suzanne Hill, Peter Kench, Yobelli Jimenez

**Affiliations:** 1 Discipline of Medical Imaging Science, School of Health Sciences, Faculty of Medicine and Health, University of Sydney, Sydney 2006, Australia; 2 I-MED Radiology Network, Sydney 2000, Australia; 3 School of Health Sciences, Western Sydney University, Sydney 2751, Australia

**Keywords:** infection prevention and control, sonographers, survey, ultrasound, radiology

## Abstract

Background/Objectives: Infection prevention and control (IPC) is fundamental to sonography practice. This study aimed to describe IPC responsibilities, sources of IPC information, and self-reported adherence to standard and transmission-based precautions among Australian sonographers. In addition, the study aimed to examine relationships between workplace characteristics and knowledge, attitudes, and practice (KAP) toward IPC. Methods: A cross-sectional online survey was distributed using convenience sampling between January to June 2025 to sonographers working in Australia. The survey questions were developed from IPC guidelines and reviewed by two practicing sonographers for face and content validity. Survey responses were analysed descriptively. Differences in demographic, workplace, and KAP variables were examined using Pearson’s chi-square and Mann–Whitney U tests, and associations between KAP domains were assessed using Spearman’s correlation. Statistical significance was set at *p* < 0.05. Qualitative responses regarding barriers to IPC adherence were analysed using deductive content analysis. Results: A total of 109 sonographers completed the survey. Participants reported working in public (37.6%) and private (62.4%) settings. Average KAP scores were high and comparable across public and private settings (knowledge: 96.0% ± 6.8% vs. 94.3% ± 11.5%; attitudes: 90.7% ± 12.3% vs. 85.4% ± 16.6%; practice: 89.3% ± 9.5% vs. 88.4% ± 13.9%) and there were no significant differences in scores between public and private workplaces (*p* > 0.05). Moderate correlations were observed between knowledge and attitudes (rho = 0.350, *p* < 0.001) and attitudes and practice (rho = 0.308, *p* = 0.001). Staffing and IPC resources were not associated with KAP scores. Use of policies and guidelines was the only factor associated with higher knowledge scores (*p* = 0.042). Participants reported high adherence to standard precautions was common, though time pressure, interruptions, and emergency situations were reported barriers. For transmission-based precautions, communication issues, particularly lack of awareness of required precautions, were the primary barrier, alongside time pressures. Conclusions: This study found that surveyed Australian sonographers have strong IPC KAP across clinical settings. Access to modality-specific IPC policies and guidelines appears critical for supporting knowledge and promoting IPC adherence.

## 1. Introduction

Infection prevention and control (IPC) is a fundamental component of healthcare delivery and an essential strategy for reducing healthcare-associated infections and promoting patient safety [1,2]. Effective IPC practices protect patients, healthcare workers, and healthcare systems by limiting the transmission of infectious agents across diverse clinical settings. Sonography practice presents distinctive IPC challenges compared to other health and medical imaging modalities, particularly in relation to sonography equipment such as transducers, which come into direct contact with patients [1,3,4,5]. Unlike modalities such as conventional radiography, computed tomography (CT) and magnetic resonance imaging, sonography examinations typically involve prolonged direct patient contact, frequent handling of equipment between patients, the application of gel, and repeated contact of transducers with intact and non-intact skin or mucous membranes [3,4,5]. Consequently, varying levels of IPC processes are recommended for safe sonography practice, depending on the nature of the sonographic examination (e.g., invasive versus non-invasive), anatomical site and the patient’s infection risk [5,6,7,8].

To support IPC in sonography, international health organisations and professional bodies have developed guidelines and recommendations [5,6,7,8,9,10]. In the Australian context, IPC guidance is provided by national organisations, including the Australasian Society for Ultrasound in Medicine (ASUM) and the Australasian Sonographers Association (ASA) [6,10]. Despite the availability of these resources, a small number of reports provide examples of inconsistent IPC knowledge, attitudes and practice (KAP) among sonographers, with variations observed across clinical settings, geographic regions, and within high-risk and invasive procedures [2,4,11,12,13]. Identified concerns in IPC practice include inadequate transducer disinfection, inconsistent hand hygiene, and suboptimal use of personal protective equipment (PPE), particularly during time-pressured clinical workflows or limited resource environments [11,12,13]. International surveys have reported considerable variation in ultrasound probe reprocessing, disinfection compliance, and use of protective barriers across healthcare settings [2,4]. Australian evidence is particularly sparse, with one ASUM survey identifying substantial variation in transducer disinfection practices among ultrasound practitioners [11]. Additional concerns have been highlighted by a healthcare-associated outbreak of *Burkholderia cenocepacia* linked to contaminated ultrasound gel used during ultrasound-guided procedures [12] and by regulatory alerts addressing the risks associated with inadequate ultrasound probe decontamination [13]. Although previous studies have examined selected aspects of IPC practice among sonographers [2,4,11], contemporary Australian evidence evaluating sonographers’ IPC knowledge, attitudes and self-reported practices remain scarce, and little is known about how workplace characteristics influence these outcomes.

Understanding IPC KAP among health professionals can help identify knowledge gaps, inform workforce development and quality improvement initiatives, support implementation of evidence-based policies and ultimately improve patient and staff safety [14,15]. Hence, the primary aim of this study was to examine IPC knowledge, attitudes and self-reported practice, responsibilities and sources of information among Australian sonographers. A secondary aim was to explore associations between these outcomes and workplace characteristics, including practice setting, year of professional experience, IPC training, access to IPC resources, and the availability of workplace IPC support mechanisms.

## 2. Materials and Methods

### 2.1. Study Design and Ethical Approval

An online survey based on the *Australian Guidelines for the Prevention and Control of Infection in Healthcare* [16] was tailored to ultrasound settings using survey tools from similar studies [14,17]. The survey questions address universal IPC principles, with some ultrasound specific context included. The draft survey items were reviewed by two experienced sonographers to assess content relevance, clarity, and appropriateness for sonography practice. Following feedback, minor amendments were made to item wording and formatting to improve readability and ensure consistency with contemporary sonography practice. The survey was developed using the Research Electronic Data Capture (REDCap) [18] platform and distributed via email invitation to Medical Imaging departments across Australia, as well as through online advertising via the Australian Sonographer Accreditation Registry (ASAR). Eligible participants were qualified sonographers practising in Australia in public or private clinical settings. Students and non-clinical sonographers were not eligible to participate. Snowballing was permitted, with requests to forward the survey to colleagues who worked in ultrasound. As recruitment occurred through open distribution, online advertising, and snowball sampling, the total number of individuals who received the study invitation was unknown. Consequently the response rate could not be calculated. A formal size calculation was not performed because the study was exploratory in nature. The achieved sample size is therefore reported descriptively and should be interpreted accordingly. The survey remained open for six-months (January to June 2025). Ethical approval was granted by The University of Sydney’s Human Research Ethics Committee (Project number: 2023/493). The survey was anonymous and survey submission indicated consent and participants were required to answer all questions before progressing to the next section. Only data from participants who completed and submitted the survey were included in the analysis. Participants could elect to enter a draw to win one of four AUD$200 gift vouchers after completing the survey.

### 2.2. Data Collection

The survey consisted of four sections. Firstly, participants were asked to provide demographic data and information regarding their personal experience in their workplace. There were a total of 13 demographic questions and 27 workplace questions. Participants were asked to select their workplace type (public hospitals, private hospitals, and private practices/clinics). Workplace demographic questions included staffing, equipment, workload, as well as the availability of infection control teams, guidelines and training. Secondly, participants were asked to respond to 11 questions about their IPC knowledge, addressing hand hygiene, PPE use and equipment disinfection using True/False statements. The next section presented participants with statements focused on IPC attitude (11 statements measured on a 5-point Likert scale ranging from ‘strongly disagree’ to ‘strongly agree’) and self-reported practice (8 statements measured on a 5-point Likert scale ranging from ‘never’ to ‘always’). There were two additional practice questions related to cleaning equipment using a 7-point Likert scale with more specific timeframes, ranging from ‘between every patient’ and ‘never’. Finally, to assess perceptions of IPC risk scenarios, participants were asked to respond to the following statement: “If I was not to adhere to standard or transmission-based precautions in Ultrasound it would be when …”, gathering open-ended responses.

### 2.3. Data Analysis

Demographic data, information sources, and workplace caseload responses were analysed descriptively and presented as frequencies and percentages for each response option. Knowledge scores were calculated by assigning one point for each correct answer and zero points for incorrect responses, then calculating percentage correct responses. Attitude and practice scores were calculated by assigning one point to responses in the two most favourable categories (e.g., ‘strongly agree’ and ‘agree’, or ‘strongly disagree’ and ‘disagree’ for reverse-scored items) and zero points to all other response categories. A score of 100% represented the highest attainable score for each of the three KAP factors, while 0% represents the lowest.

Private hospital and private practice workplaces were grouped as ‘private setting’ for analysis. This grouping is based on the observation that in Australia, private radiology companies often run medical imaging departments within private hospitals, and consequently adopt similar workplace cultures, caseload expectations, training regimes and information sharing practices. Additionally, for questions with ‘Yes’, ‘No’ and ‘Unsure’ response options, ‘No’ and ‘Unsure’ were combined together during analysis. Pearson’s chi-square test was used to compare demographic and workplace data, while the Mann–Whitney U test was used to examine relationships between workplace characteristics and KAP responses. Spearman’s correlation was used to examine potential associations among KAP responses. The level of significance (*p*-value) was set at 0.05, and R version 4.5.1 was used for all statistical testing.

Open-ended responses regarding non-adherence to IPC precautions were analysed using inductive analysis [19]. Data were deemed complete when responses showed clear participant intent to answer the question using a phrase or a full sentence. One author (KA) independently coded participants’ responses into similar categories, then met regularly with all authors to reach a consensus regarding the analysis. Similar codes were grouped together into broader categories through discussion among the research team.

## 3. Results

### 3.1. Study Participants

Overall, 109 participants completed the survey and were included in the analysis. Table 1 describes participants’ characteristics and workplace demographics. Participants described their workplace as a public hospital (n = 41, 37.6%) or a private setting (n = 68, 62.4%). Private setting included private practice (n = 55, 50.5%), private hospital (n = 4, 3.7%), private practice in a private hospital (n = 8, 7.3%), and private practice in a public hospital (n = 1, 0.9%). The majority of responses came from New South Wales, Victoria, and Queensland, which are the more populous states of Australia, accounting for 78.9% of all responses. A near even representation of metropolitan (n = 62, 56.9%) and rural or remote sites (n = 47, 43.1%) was reported. The median age range of respondents was between 41 and 50 years, and 75.2% had been practising ultrasound for 10 years or more. Respondents reported working an average of 3.6 days, or a median of 4 days a week.

### 3.2. Workplace Caseload, Roles and IPC Structures

On a daily basis, the majority of respondents (73.4%) reported completing between 10 and 20 patient scans per shift. Respondents from private settings reported a median of 15 scans per shift, while those from public settings reported a median of 12 scans. Table 2 presents information about professional groups that participants commonly worked with, and the proportion of studies that involved invasive techniques, based on their workplace type. More than half of participants (56%) working in public hospitals reported nurses working alongside sonographers in the imaging department, whereas only 26% of private workplaces involved a nurse in their departments (Χ^2^(1) = 9.568, *p* = 0.002). This trend was also similar in the greater prevalence of patient transport staff in public settings (Χ^2^(1) = 6.256, *p* = 0.012). There were no significant differences in the proportion of studies requiring guided insertions of a medical device or in the proportion of participants who performed procedures requiring additional invasive techniques between respondents from private and public settings.

Regarding IPC teams, guidelines and training, a higher proportion of respondents from public settings reported having a dedicated infection control team at the workplace compared to private settings (Χ^2^(2) = 45.57, *p* < 0.001) as well as receiving training in IPC within the past 12 months (Χ^2^(4) = 10.012, *p* = 0.040) (Table 2). 

**Figure 1:**
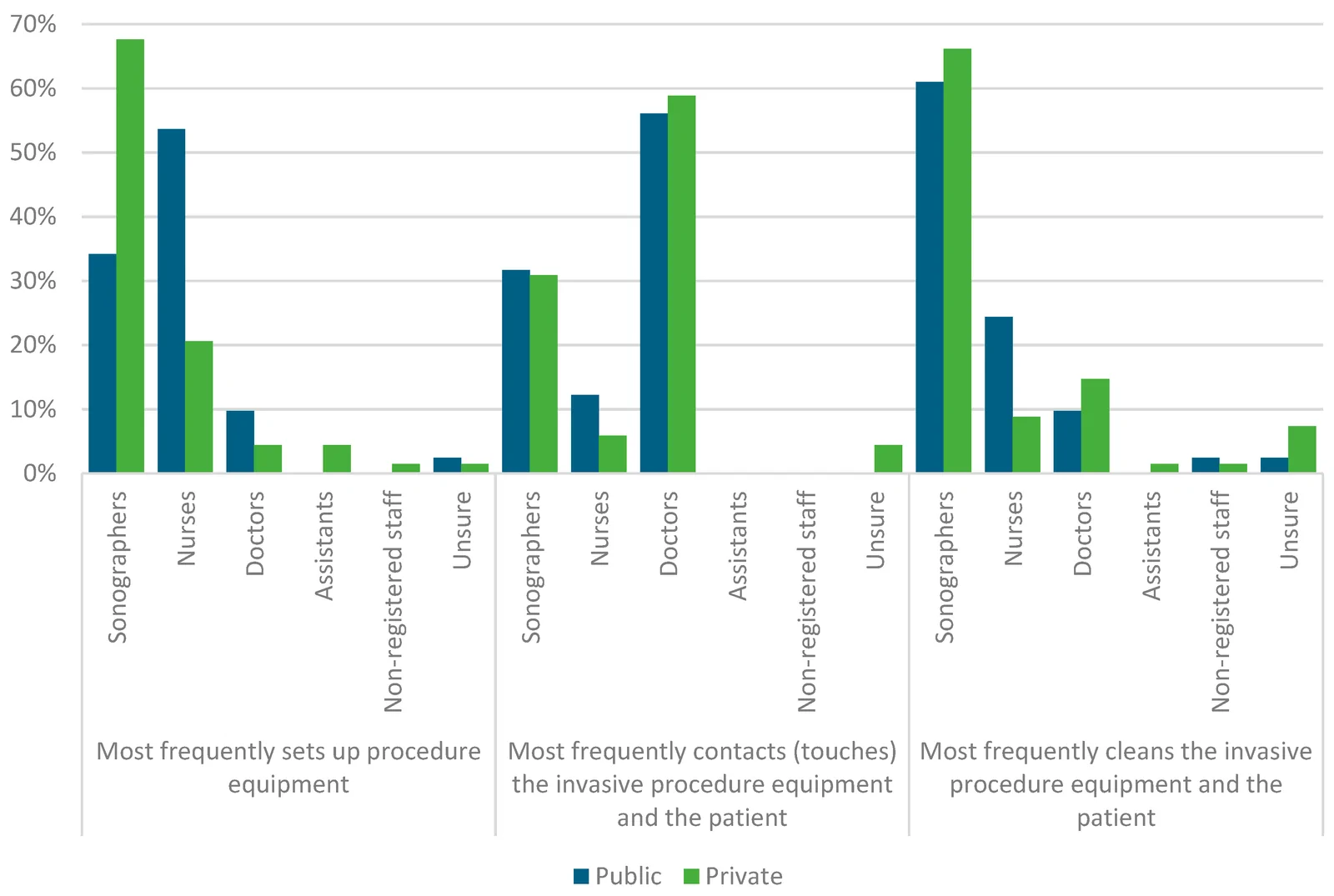
Occupational responsibilities defined by sonographers working in public (n = 41) and private (n = 68) settings, relating to which profession most frequently (1) sets up procedure equipment, (2) contacts (touches) the invasive equipment and the patient, and (3) cleans invasive procedure equipment and the patient.

Figure 1 describes the occupational responsibilities across three steps of an invasive procedure, including the preparation of procedural equipment, contact with the equipment and post-procedural cleaning. In public settings, nurses were predominantly responsible for the preparation of procedural equipment, whilst in private settings, sonographers were much more likely to set up procedural equipment and clean the equipment and the patient. Significant differences were found between private and public settings in the occupational responsibilities for setting up procedural equipment (Χ^2^(5) = 17.365, *p* = 0.004).

**Figure 2:**
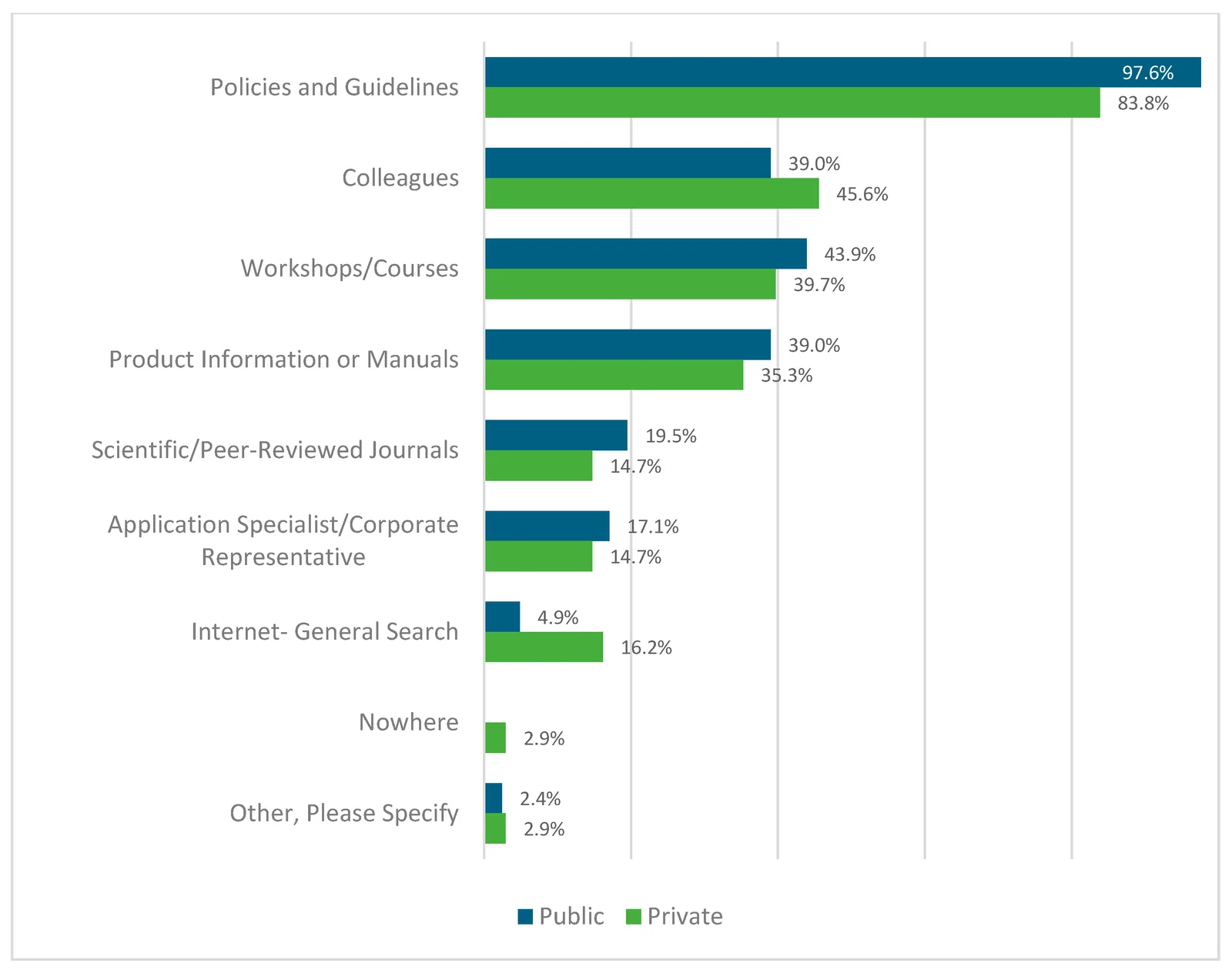
Sources of IPC information reported by study participants (n = 109), separated by responses from sonographers working in public (n = 41) or private (n = 68) settings. Participants were permitted to select multiple responses from a list of options.

Figure 2 shows the sources of IPC information reported by study participants. Most participants reported policies and guidelines as the most common source (n = 97, 89.0%), followed by workshops (n = 47, 43.1%), colleagues (n = 47, 43.1%) and peer-reviewed journal articles (n = 18, 16.5%). Respondents from public settings were more likely to use policies and guidelines as a source than those from private settings (Χ^2^(1) = 4.927, *p* = 0.026). No statistically significant differences were found between public and private settings regarding departmental IPC support, including the ease of staying home if sick, the availability of IPC products, and an emphasis on the importance of IPC.

### 3.3. Knowledge, Attitudes and Practice (KAP)

**Figure 3:**
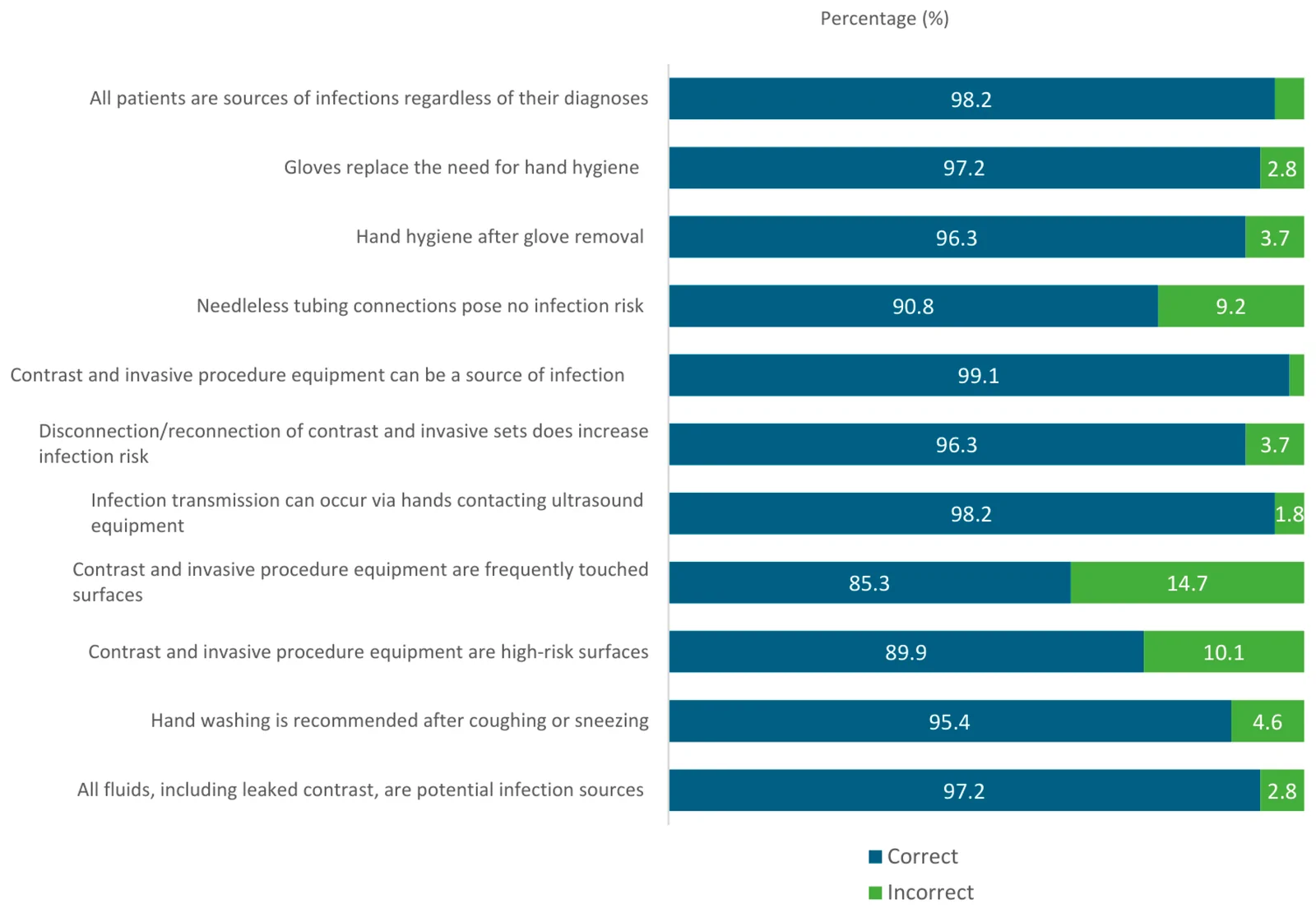
Percentage of correct and incorrect responses to statements regarding knowledge of sonographers relating to infection, prevention and control for all study participants (n = 109).

**Figure 4:**
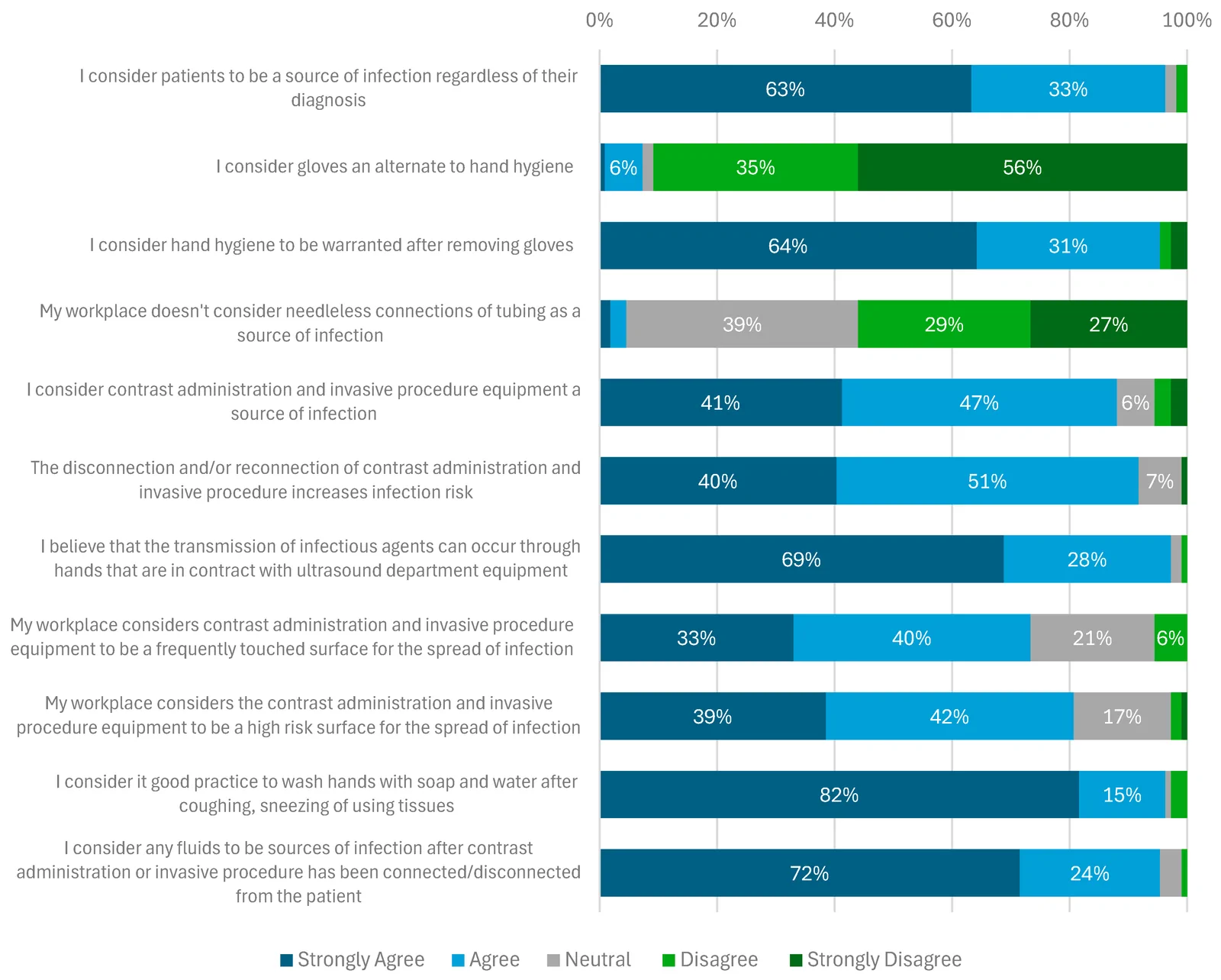
Responses to ‘Attitudes’ domain statements relating to infection, prevention and control shown as percentages of total responses for each item (n = 109).

**Figure 5:**
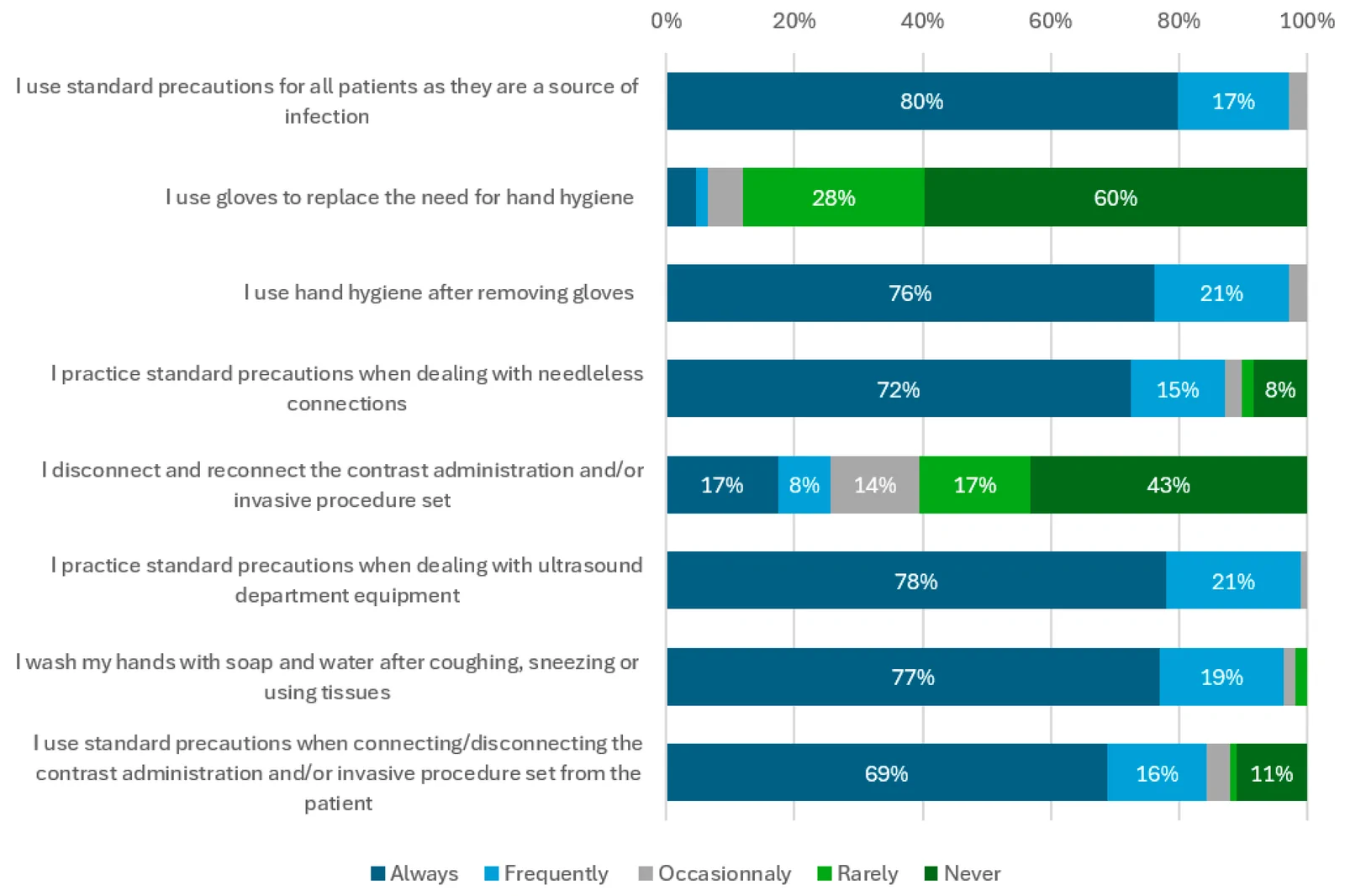
Responses to ‘Practice’ domain statements relating to infection, prevention and control (IPC), shown as percentages of total responses for each item (n = 109). Data relates to self-reported practice.

Participants’ responses to ‘Knowledge’, ‘Attitude’, and ‘Practice’ domains are presented in Figure 3, Figure 4, and Figure 5 respectively. Overall, participants from both public and private settings scored comparable averages of 94.9% ± 10% in knowledge (96.0% ± 6.8% vs. 94.3% ± 11.5%), 87.4% ± 15.2% in attitude (90.7% ± 12.3% vs. 85.4% ± 16.6%) and 88.8% ± 12.4% in practice (89.3% ± 9.5% vs. 88.4% ± 13.9%), respectively. As shown in Figure 3, the statement with the highest proportion of correct answers for knowledge related to contrast and invasive procedure equipment being a source of infection (99.1% correct responses) and the least percentage of correct responses related to the statement regarding contrast and invasive procedure equipment being a high-risk surface (85.3% correct responses). In the ‘Attitudes’ domain, the highest agreement percentage related to the statement “I believe that transmission of infectious agents can occur through hands in contact with ultrasound equipment”, with 97.2% of participants responding “strongly agree” or “agree”, as shown in Figure 4. In the ‘Practice’ domain, two statements reported the highest combination of “Always” or “Frequently” (97.2%): “I use standard precautions for all patients as they are a source of infection” and “I practice standard precautions when dealing with ultrasound department equipment”, as shown in Figure 5. Due to the data collection method, practice frequency was self-reported.

Significant moderate correlations were found between knowledge and attitudes (rho = 0.350, *p* <0.001), as well as attitudes and practice (rho = 0.308, *p* = 0.001). Using the Mann–Whitney U test, there were no significant relationships between workplace setting and KAP scores, both overall and individually, for knowledge, attitudes or self-reported practice domains. The presence of nurses, patient transport staff, a departmental IPC team or guidelines also had no statistically significant effect on KAP scores individually and overall. The use of policies and guidelines was the only information source that influenced knowledge scores across all respondents (W = 791.5, *p*-value = 0.042).

### 3.4. Non-Adherence to IPC

**Figure 6:**
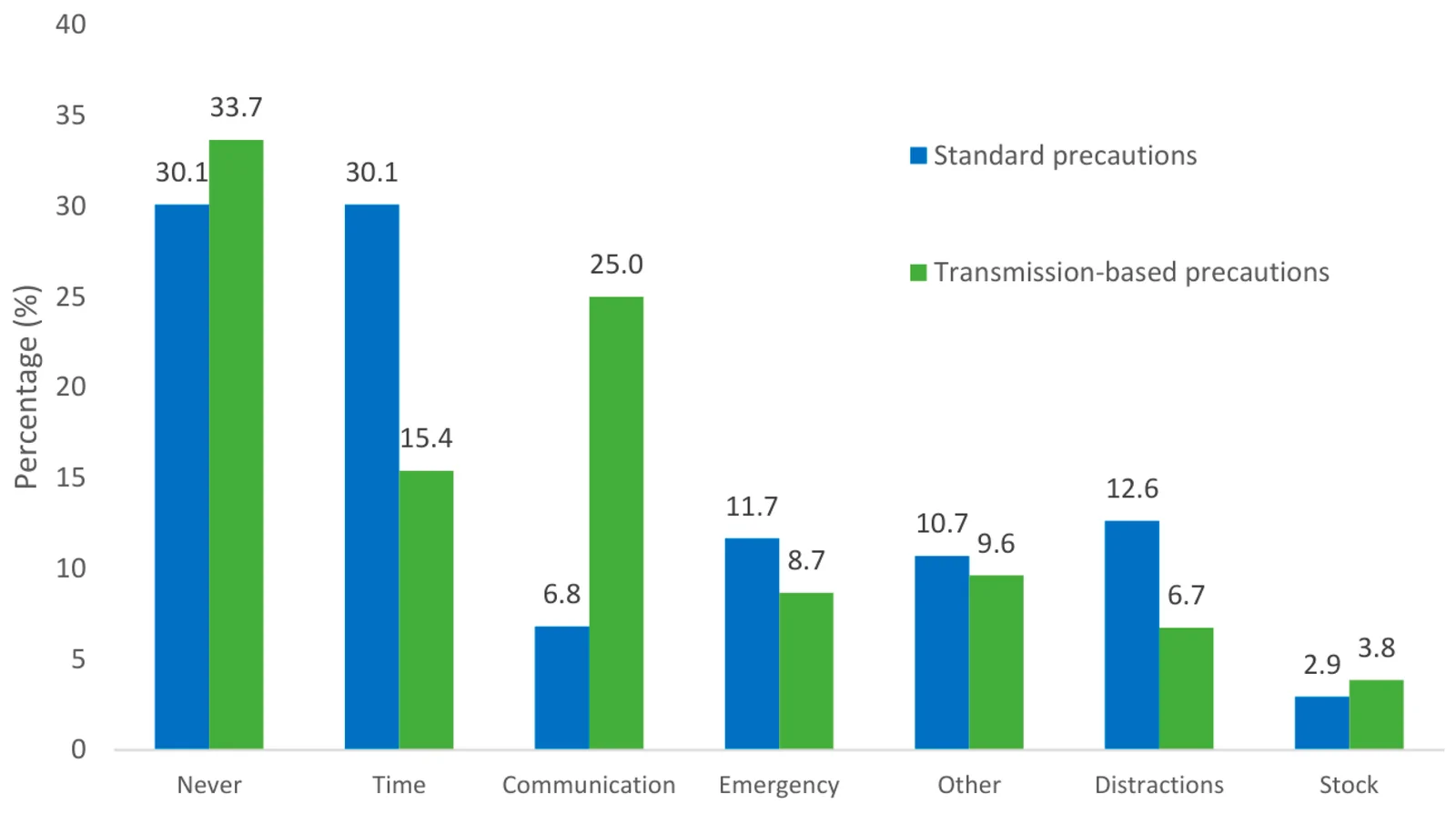
Percentage of responses to clinical scenarios when study participants would not adhere to standard (n = 103 responses) and transmission-based precautions (n = 104 responses).

Of the 109 survey responses, 103 and 104 participants answered the open-ended questions regarding clinical scenarios in which they would not adhere to standard precautions and transmission-based precautions, respectively (Figure 6). For standard precautions, 103 participants provided a total of 108 reasons. The majority of participants (n = 31, 30.1%) stated they would, without exception, always adhere to standard precautions, whilst the highest contributor for non-adherence was attributed to time pressures and stress with 31 (30.1%) participants providing responses such as “It is likely when I am being rushed or feeling pressured” and “Running late or fitting in extra patients”. One respondent also described that maintaining IPC adherence can cause them to run late. Other barriers to compliance to standard precautions were attributed to interruptions and distractions, including reports of being “distracted while with a patient by an external factor” or “when my infection control process is interrupted” encapsulating 13 responses. Respondents also described emergency situations as a barrier in 12 cases, such as when “we have a foetal loss”, or “in a life-threatening situation”.

Regarding transmission-based precautions, 104 participants provided a total of 107 reasons for this question. The majority (n = 35, 33.7%) stated ‘Never’ for non-adherence, with inadequate communication reported as the largest barrier to adherence, with 26 responses. Communication issues included being “unaware that precautions were required. This information is not always easily accessible”, “poor communication has led to the radiology department not being aware that the patient has precautions”, and when there is an “overlap of patients or [when] working with others, assuming someone has already cleaned”. Many participants also reported time pressures being a barrier, such as when “there were too many patients” and when they “have been double booked”. Emergency situations were also a barrier for transmission-based precautions including when an “accident occurs in the procedure”, “there is an emergency,” and in “urgent cases”.

## 4. Discussion

IPC is fundamental to patient safety in medical imaging, particularly in ultrasound practice where close patient contact and the use of shared equipment create opportunities for infection transmission. This study provides a comprehensive report on workplace characteristics of sonographers working in Australia, as well as on sonographers’ knowledge, attitudes and self-reported practice regarding IPC. Sonographers in private settings reported a slightly higher workload than those in public hospitals, likely due to differences in service models, patient characteristics, and staffing structures. High examination volumes may be linked to IPC non-compliance in this study, as a proportion of study participants (n = 31/103, 30.1%) indicated that time pressure was a reason when they would not adhere to standard precautions. This result is similar to high IPC-risk scenarios reported by CT radiographers. In the CT environment, CT radiographers identified staffing issues, procedural complications, trauma cases, and emergency settings as barriers to IPC compliance [20]. Similarly, participants in the current study described sonography-specific scenarios in which patient care priorities, such as managing foetal loss, influenced IPC-related decision making.

Sonographers are also required to work effectively with other professions, with some differences in multidisciplinary support observed among participants in this study. More sonographers in public hospitals reported working alongside nurses and patient transport staff compared to those in private practices. Shared occupational responsibilities may reflect the higher complexity of patient caseloads for invasive procedures in public hospitals. Research has shown that among CT radiographers and wider health care teams, the involvement of additional staff may increase opportunities for shared responsibility, but may also introduce additional transmission pathways or breaks in communication between individuals or professional groups [20,21,22]. The current study found that ineffective communication within teams poses challenges to IPC compliance among sonographers. Most sonographers were involved in invasive techniques and procedures, such as biopsies, with varying responsibilities, including setting up equipment, patient contact and equipment handling during the procedure, and post-procedure cleaning. Given that invasive sonography procedures increase the risk of infection transmission, adequate knowledge and practice of aseptic techniques are imperative for sonographers. There is an inherent responsibility of sonographers to possess appropriate knowledge and attitudes toward IPC, to ensure safe and effective practice [9,16].

The study demonstrates high levels of IPC knowledge, positive attitudes and self-reported practices among Australian sonographers across both public and private settings. No significant differences were observed in KAP domain scores between public and private practice settings, nor the presence of nurses or transport staff, departmental IPC teams or local guidelines. This contrasts with earlier research in CT environments, which reported higher attitudes and self-reported practice among radiographers working in public hospitals and in settings with dedicated IPC teams [14]. A possible reason is the ceiling effect created by uniformly high knowledge scores, which may reduce the ability of the survey tool to discriminate between workplace-level settings. This finding may also suggest that IPC knowledge has become embedded within sonography practice, regardless of the organisational context. Sonographers also have access to existing IPC guidelines and recommendations specific to the sonography context, which is lacking in other medical imaging settings [23]. Nonetheless, differences in KAP among professional groups working in medical imaging settings have been reported previously. For example, a previous study found that nurses working in the radiology department scored significantly higher on IPC practice than radiographers, even though knowledge scores were similarly high [14].

Overall, knowledge, attitudes and self-reported practice scores were above 85% in all domains, with knowledge scoring highest, followed by self-reported practice and attitudes. These findings are consistent with reported IPC knowledge among CT radiographers and other health professionals [14,15], where positive correlations have been observed between understanding IPC principles and willingness to follow best practice [14]. High self-reported compliance in the current study may reflect sonographers’ awareness of fundamental IPC behaviours, including hand hygiene, which is consistently reported as a cornerstone of patient safety in medical imaging environments, although the proximity of patient contact could also affect sonographers’ awareness of IPC and, subsequently, behaviour [9]. However, self-reported measures are susceptible to social desirability bias and may overestimate actual compliance. Observational studies would be valuable in determining whether reported behaviours are reflected in routine clinical practice. Furthermore, previous studies have reported discrepancies between knowledge and practice among radiographers in Namibia [24], suggesting that high self-reported practice scores should be interpreted with caution.

Access to policies and guidelines was the only information source associated with significantly higher knowledge scores. Previous studies have highlighted that inconsistency in IPC guidelines may undermine practitioner confidence and compliance [4,23]. However, the existence of ultrasound-specific guidelines published by professional bodies [5,6,7,8,9,10] supports the importance of standardised protocols for specific sonography equipment, including transducer disinfection, gel handling, and hand hygiene, particularly given documented outbreaks in sonography [12,13,25]. Colleagues as sources of information were less common among sonographers in this study, in contrast to CT radiographers in previous studies, where colleagues were reported to be the most common source of information among qualified radiographers [14].

Open-ended responses in this study provided insight into contextual and behavioural factors in sonography practice, and a substantial proportion of respondents reported they would never intentionally deviate from standard or transmission-based precautions. The scenarios that participants reported compromised IPC adherence were due to time pressures, communication failures, interruptions and emergencies. These findings suggest that non-adherence is driven less by deficiencies in knowledge and more by competing clinical demands and organisational factors. Consequently, strategies aimed solely at education may be insufficient without concurrent attentions to workflow design, staffing, communication processes, and workplace culture.

### Limitations

The main limitations of this study are response bias from self-reported individual practices and attitudes, and self-selection, whereby only interested participants completed the survey. In addition, because IPC practices were self-reported, social desirability bias may have resulted in an overestimation of actual IPC adherence. Observational studies may provide a more accurate assessment of clinical practice behaviours. Given that this data was collected only from a relatively small number of Australian sonographers, the results are unlikely to be widely generalisable to other geographic locations where professional roles and healthcare systems can vary widely. However, participants were recruited from both metropolitan and rural workplaces, and many held senior positions within the profession, providing perspectives from a range of practice settings and levels of experience. Although the survey underwent expert review by two experienced sonographers to establish face validity, formal psychometric validation and reliability testing were not performed. Consequently, the findings should be interpreted with consideration of the potential limitations of the survey instrument. Private hospitals and private practices were combined into a single private sector category for analysis. Although this approach increased statistical power, it may have masked differences in IPC practices arising from variations in patient acuity, inpatient services, and procedural complexity across private healthcare settings.

## 5. Conclusions

Australian sonographers who participated in this study demonstrate high levels of IPC knowledge, positive attitudes, and strong self-reported adherence to recommended practices across a range of clinical settings. However, the findings indicate that maintaining effective IPC extends beyond individual competence, with workplace pressures, communication challenges, and clinical demands influencing adherence to precautions. Sonography-specific policies and professional guidelines remain important resources for promoting safe practice and reducing the risk of infection transmission in ultrasound care. These findings highlight that effective IPC in sonography requires both knowledgeable practitioners and workplace systems that enable consistent adherence to recommended precautions.

## Figures and Tables

**Table 1:** Study participants’ demographic data of study participants.

Demographic		Public (n = 41)	Private (n = 68)	Total (n = 109)
Gender	Female	32 (78.0%)	58 (85.3%)	90 (82.6%)
	Male	9 (22.0%)	10 (14.7%)	19 (17.4%)
Age (years)	20–25	0 (0%)	1 (1.5%)	1 (0.9%)
	26–30	1 (2.4%)	2 (2.9%)	3 (2.8%)
	31–35	3 (7.3%)	8 (11.8%)	11 (10.1%)
	36–40	7 (17.1%)	7 (10.3%)	14 (12.8%)
	41–50	13 (31.7%)	20 (29.4%)	33 (30.3%)
	51+	17 (41.5%)	30 (44.1%)	47 (43.1%)
Education	Undergraduate (Bachelor’s degree)	3 (7.3%)	1 (1.5%)	4 (3.7%)
	Postgraduate (Sonography)	17 (41.5%)	38 (55.9%)	55 (50.5%)
	Postgraduate (Graduate Certificate, Masters)	16 (39.0%)	26 (38.2%)	42 (38.5%)
	PhD	5 (12.2%)	3 (4.4%)	8 (7.3%)
Registration	Yes	41 (100%)	67 (98.5%)	108 (99.1%)
	No	0 (0.0%)	1 (1.5%)	1 (0.9%)
Years in profession	1–5 years	1 (2.4%)	5 (7.4%)	6 (5.5%)
	6–10 years	3 (7.3%)	6 (8.8%)	9 (8.3%)
	10+ years	37 (90.3%)	57 (83.8%)	94 (86.2%)
Years in Sonography profession	<1 year	0 (0.0%)	2 (2.9%)	2 (1.8%)
	1–5 years	5 (12.2%)	7 (10.3%)	12 (11.0%)
	6–10 years	3 (7.3%)	10 (14.7%)	13 (12%)
	10+ years	33 (80.5%)	49 (72.1%)	82 (75.2%)
Position	General	7 (17.1%)	18 (26.5%)	25 (22.9%)
	Senior	20 (48.8%)	32 (47.0%)	52 (47.7%)
	Section Chief: Ultrasound	14 (34.2%)	13 (19.1%)	27 (24.8%)
	Other	0 (0.0%)	5 (7.4%)	5 (4.6%)
State	New South Wales	19 (46.3%)	31 (45.6%)	50 (45.9%)
	Victoria	7 (17.1%)	12 (17.6%)	19 (17.4%)
	Queensland	5 (12.2%)	12 (17.6%)	17 (15.6%)
	Western Australia	4 (9.8%)	3 (4.4%)	7 (6.4%)
	South Australia	2 (4.9%)	7 (10.3%)	9 (8.3%)
	Australian Capital Territory	3 (7.3%)	3 (4.4%)	6 (5.5%)
	Tasmania	1 (2.4%)	0 (0.0%)	1 (0.9%)
Region	Metropolitan	23 (56.1%)	39 (57.4%)	62 (56.9%)
	Rural	18 (43.9%)	29 (42.6%)	47 (43.1%)

**Table 2:** Participant workload, professional groups and techniques by workplace type (n = 109).

Roles, Caseload and Workplace Structures		Public (n = 41)	Private (n = 68)	Total (n = 109)
Oher professions that routinely work in the department	Doctors	22 (53.7%)	38 (55.9%)	60 (55.0%)
	Nurses	23 (56.1%)	18 (26.5%)	41 (37.6%)
	Radiographers	9 (22.0%)	19 (27.9%)	28 (25.7%)
	Non-Registered Staff	4 (9.8%)	16 (23.5%)	20 (18.3%)
	Assistants	7 (17.1%)	8 (11.8%)	15 (13.8%)
	Patient transport staff	10 (24.4%)	5 (7.4%)	15 (13.8%)
	Medical Physicists	1 (2.4%)	1 (1.5%)	2 (1.8%)
Average number of studies completed on a typical shift	≤10	17 (41.4%)	9 (13.2%)	26 (23.9%)
	>10 and ≤20	22 (53.7%)	58 (85.3%)	80 (73.4%)
	>20	2 (4.9%)	1 (1.5%)	3 (2.7%)
Procedures performed with contrast and/or guided insertions of invasive medical devices.	Yes	23 (56.1%)	38 (55.9%)	61 (56.0%)
	No	18 (43.9%)	29 (42.6%)	47 (43.1%)
	Unsure	0 (0%)	1 (1.5%)	1 (0.9%)
Studies involving invasive techniques in addition to standard ultrasound imaging (Average percentage)	0–25%	32 (78.1%)	50 (73.5%)	82 (75.3%)
	26–50%	6 (14.6%)	11 (16.2%)	17 (15.6%)
	50–75%	0 (0%)	2 (2.9%)	2 (1.8%)
	75+%	0 (0%)	1 (1.5%)	1 (0.9%)
	Unsure	3 (7.3%)	4 (5.9%)	7 (6.4%)
Clinical site has dedicated infection control team	Yes	37 (90.2%)	16 (23.5%)	53 (48.6%)
	No	3 (7.3%)	40 (58.8%)	43 (39.5%)
	Unsure	1 (2.5%)	12 (17.7%)	13 (11.9%)
Clinical site has IPC policies and guidelines for all procedures	Yes	38 (92.7%)	60 (88.2%)	98 (89.9%)
	No	0 (0.0%)	3 (4.4%)	3 (2.8%)
	Unsure	3 (7.3%)	5 (7.4%)	8 (7.3%)
IPC training or orientation	In the past 12 months	34 (82.9%)	39 (57.3%)	73 (67.0%)
	In the past 1–3 years	3 (7.3%)	17 (25.0%)	20 (18.3%)
	In the past 4–5 years	0 (0.0%)	4 (6%)	4 (3.7%)
	More than 5 years ago	3 (7.3%)	4 (6%)	7 (6.4%)
	Never	1 (2.5%)	4 (6%)	5 (4.6%)

IPC: Infection prevention and control.

## Data Availability

The raw data supporting the conclusions of this article will be made available by the authors on request due to ethical and governance considerations. The study’s ethics approval permits use of the data by the approved research team for the purposes outlined in the approved protocol. Any requests for access will be assessed to ensure the proposed use is consistent with participant consent and ethical requirements. Where required, additional ethics approval may need to be obtained before data can be shared.
